# Jasmonate-Dependent Response of the Flower Abscission Zone Cells to Drought in Yellow Lupine

**DOI:** 10.3390/plants11040527

**Published:** 2022-02-15

**Authors:** Agata Kućko, Aleksandra Bogumiła Florkiewicz, Magdalena Wolska, Jakub Miętki, Małgorzata Kapusta, Krzysztof Domagalski, Emilia Wilmowicz

**Affiliations:** 1 Department of Plant Physiology, Institute of Biology, Warsaw University of Life Sciences-SGGW (WULS-SGGW), Nowoursynowska 159 Street, 02-776 Warsaw, Poland; 2 Department of Plant Physiology and Biotechnology, Nicolaus Copernicus University, 1 Lwowska Street, 87-100 Toruń, Poland; a.florkiewicz21@gmail.com (A.B.F.); magda_w@umk.pl (M.W.); 285417@stud.umk.pl (J.M.); emwil@umk.pl (E.W.); 3 Department of Plant Cytology and Embryology, University of Gdańsk, 59 Wita Stwosza, 80-308 Gdańsk, Poland; malgorzata.kapusta@biol.ug.edu.pl; 4 Department of Immunology, Nicolaus Copernicus University, 1 Lwowska Street, 87-100 Toruń, Poland; krydom@umk.pl

**Keywords:** abscission zone, CORONATINE INSENSITIVE1, drought, flower abscission, JASMONATE RESISTANT1, jasmonic acid, jasmonates, lipoxygenase, phospholipase D

## Abstract

Lipid membranes, as primary places of the perception of environmental stimuli, are a source of various oxygenated polyunsaturated fatty acids—oxylipins—functioning as modulators of many signal transduction pathways, e.g., phytohormonal. Among exogenous factors acting on plant cells, special attention is given to drought, especially in highly sensitive crop species, such as yellow lupine. Here, we used this species to analyze the contribution of lipid-related enzymes and lipid-derived plant hormones in drought-evoked events taking place in a specialized group of cells—the flower abscission zone (AZ)—which is responsible for organ detachment from the plant body. We revealed that water deficits in the soil causes lipid peroxidation in these cells and the upregulation of phospholipase D, lipoxygenase, and, concomitantly, jasmonic acid (JA) strongly accumulates in AZ tissue. Furthermore, we followed key steps in JA conjugation and signaling under stressful conditions by monitoring the level and tissue localization of enzyme providing JA derivatives (JASMONATE RESISTANT1) and the JA receptor (CORONATINE INSENSITIVE1). Collectively, drought-triggered AZ activation during the process of flower abscission is closely associated with the lipid modifications, leading to the formation of JA, its conjugation, and induction of signaling pathways.

## 1. Introduction

Jasmonates, among them jasmonic acid (JA), as well as its derivatives, such as methyl jasmonate (MeJA), and jasmonoyl-L-isoleucine (JA-Ile), are a diverse group of galactolipid-derived phytohormones ubiquitously distributed in plant cells [1]. Particular members of JAs exhibit various activities in the physiological, metabolic, developmental, and defensive processes in different species [2,3]. Additionally, JAs are known to promote senescence [4,5]. There are also several reports indicating their involvement in organ separation processes, e.g., in *Arabidopsis thaliana* [6,7], *Bryophyllum calycinum* [8], *Citrus sinensis* [9], *Kalanchoe blossfeldiana* [10], *Rosa chinensis* [11], and *Phaseolus vulgaris* [12]. The positive effect of JA on organ detachment was observed in *A. thaliana* [6,7], while MeJA stimulated this process in *K. blossfeldiana*, and *B. calycinum* [8,10]. JAs regulate the process of organ separation at various stages, starting with modulation of the expression of genes encoding enzymes that are involved in the modification and rearrangement of cell walls (e.g., polygalacturonase, pectinase, xylanase, pectin methylesterase), through the formation of a secondary abscission zone [8], and finishing with their involvement in the degradation of polysaccharides in the abscission zone (AZ)—a place of organ shedding [10]. Furthermore, JAs’ role is even more complicated, given their interaction with other phytohormones, e.g., ethylene (ET) or auxins, as described in *A. thaliana* and *C. sinensis* [13,14]. In turn, the independent action of JAs has been observed in species such as *Oryza sativa* [15], *Zea mays* [16], *Fagopyrum tataricum* [17], and *Agropyron cristatum* [18]. Considering that JAs are a large, heterogeneous group of phytohormones, which differ in their chemical structure, spectrum of activity, and possible interaction with other hormones, studies regarding JAs’ role in abscission-related processes are necessary. Such research is important especially in crops, such as *Fabaceae*. A key factor affecting the organ separation in these species is soil drought. Water deficit causes organ AZ activation, leading to extensive, and more importantly, preliminary abortion [19,20,21]. It has been recently pointed out that flower abscission in *Lupinus luteus*, economically important *Fabaceae*, is accelerated by JAs and is accompanied by lipid modification specifically in AZ cells [22]. Given that JAs participate in the response of a plant to water deficit, in this paper, we answer a question: whether the soil drought stress can affect the JAs-related events taking place in extremely sensitive tissue—that being flower AZ.

The consequence of drought is the generation of oxidative stress in the plant. Reactive oxygen species (ROS) accumulated in high concentrations cannot be detoxified by enzymatic or non-enzymatic repair systems and might reorganize cell membranes, especially lipid components [19,21,23]. It has been demonstrated many times that water stress is correlated with an increased level of malondialdehyde (MDA) [24,25]. Concomitantly, the activity of enzymes responsible for cell membrane remodeling, such as phospholipase D (PLD) and lipoxygenase (LOX), is upregulated [25]. These proteins are the first enzymes in the JAs’ biosynthesis pathways at the same time [26]. Increased mRNA levels of the PLD gene and activity of PLD was observed in *A. thaliana* [27], sweet orange [28], *Craterostigma plantagineum* [29], *Petunia* [7], *Vigna radiata* [30], *Z. mays* [31], and *Arachis hypogaea* [32] under drought. In turn, accumulation of *LOX* mRNA evoked by water deficit has been demonstrated in *Capsicum annuum* L. [33], *P. vulgaris* [34], *Panax ginseng* [35], *Diospyros kaki* [36], *Setaria italica* [37], *Cucumis melo* [38], *Solanum lycopersicum* [39], *Agrostis stolonifera* [40], and *Triticum aestivum* [41]. LOX-encoding lipoxygenases catalyze the conversion of α-linolenic acid to (13S)-hydroperoxy-octadecatrienoic acid (13-HPOT), which is further subjected to processes of oxidation, cyclization, and acyl chain shortening, to finally form JA [42,43]. Studies made on *Triticum aestivum* [14], *A. thaliana* [44,45], *Gossypium hirsutum* [3], and *Citrus Paradisi* × *Poncirus Trifoliata* [46] have proven an existing correlation between a high endogenous level of JA and the increased transcriptional activity of *LOX* genes when plants were subjected to drought. Under such stressful conditions, JAs, mainly JA and MeJA, positively influence the level of osmoprotectants, including proline, betaine, flavonoids, sugars, and phenolic compounds [47]. These hormones regulate the functioning of antioxidant systems and determine the intensity of photosynthesis by regulation of stomata opening [47]. Other JAs derivatives also exhibit biological activity. For instance, JA conjugate with isoleucine (JA-Ile), which can be biosynthesized in a reaction catalyzed by JASMONATE RESISTANT (JAR) proteins, similarly to other amino acid derivatives [48]. During stress, the proper functioning of JAs’ signal transduction pathway is just as important as their biosynthesis. In drought-treated rice, the transcriptional repressor protein—JASMONATE ZIM DOMAIN protein (OsJAZ1)—inhibits both JAs, as well as abscisic acid (ABA) signal transduction pathways, and consequently reduces the plant’s tolerance to water deficit [44,45]. Generally, once JAs are recognized by a receptor—F-box protein COI1 (CORONATINE-INSENSITIVE 1)—then, various members of JAZ proteins are targeted for ubiquitination, and subsequently subjected to proteasomal degradation [49]. COI1 is a part of a Skp/Cullin/F-box complex (SCF^COI1^) that functions as a ubiquitin ligase [50].

In the presented paper, we used molecular, biochemical, immunocytochemical, and instrumental analyses to investigate flowers’ AZ-specific response to soil drought stress, related to JAs. First, we measured MDA content as a marker of lipid peroxidation. Next, we analyzed the impact of drought on two JA biosynthesis enzymes (PLD and LOX), followed by JA detection. Finally, we checked the appearance of JAR and COI1 under water deficit conditions. We provide evidence that JAs are connected to flower abscission processes evoked by drought, which has a strong impact on organ separation.

## 2. Results

### 2.1. Drought Changes MDA Level in Flower AZ of Lupine

**Figure 1:**
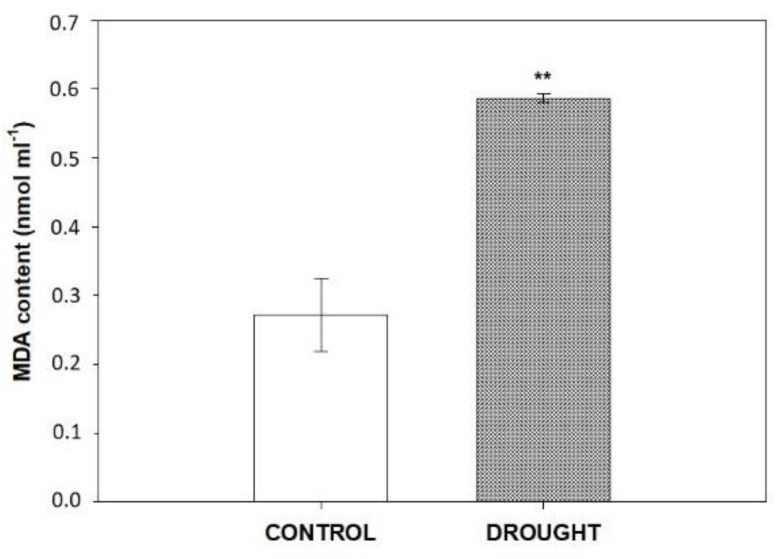
Effect of soil drought on the malondialdehyde (MDA) level in the flower abscission zone (AZ) of *Lupinus luteus*. Flower AZ was excised from plants subjected to 2 weeks of drought (25% water holding capacity, WHC) or lupines growing under optimal moisture (70% WHC, control). Results are presented as means ± SE (*n* = 3). Asterisks indicate a significant difference (Student’s *t*-test: ** *p* < 0.01).

As we have previously shown, ROS accumulate during drought-activated AZ of yellow lupine flowers [21]. Oxidative stress evoked by ROS may lead to lipid peroxidation, which could be measured by analysis of MDA [51]. Here, to better characterize the effect of the water deficit in the soil on the destabilization of the lipid bilayer in AZ of lupine flowers, we first determined the MDA level (Figure 1). As Figure 1 shows, MDA content increased two times in AZ tissues from stressed plants in comparison to AZ from non-abscised flowers (control).

### 2.2. Drought Induces the Appearance of Phospholipase D (PLD) in Flower AZ of Lupine

**Figure 2:**
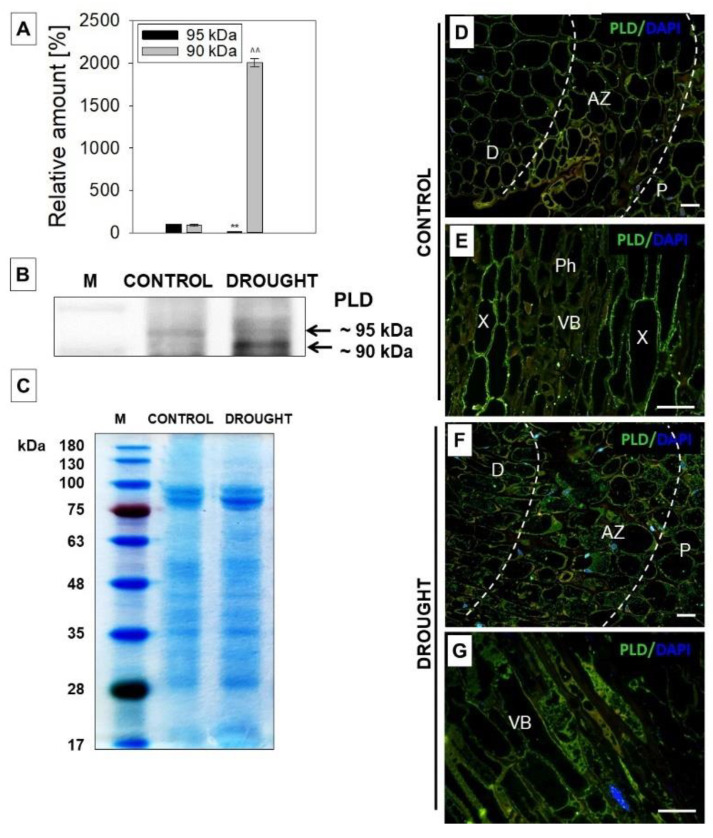
The level of phospholipase D (PLD) in the flower abscission zone (AZ) of *Lupinus luteus* is strongly affected by soil drought. For the analyses, flowers’ AZ fragments were collected on the 48th day of cultivation from drought-treated lupines (25% water holding capacity, WHC) or control plants (70% WHC). For details, see the Material and Methods section. Western blot analysis was performed with an anti-PLD antibody and revealed the presence of two reactive bands of ~90 kDa and 95 kDa (**B**). These bands were scanned, and densitometry data were generated (100% was set for the control (**A**). Results on the chart are presented as means ± SE. A significant difference in AZ from drought-treated plants in comparison to control for the isoform of ~95 kDa are ** *p* < 0.01, while for the isoform of ~90 kDa are ^^^^ *p* < 0.01. Coomassie brilliant blue-stained SDS-PAGE gel (**C**). Molecular mass marker (M), sizes are shown in kDa. Immunolocalization of PLD in flower AZ of control (**D**,**E**) and drought-stressed lupines (**F**,**G**). Green fluorescence indicates the presence of PLD, whereas blue labeling corresponds to nuclei stained with DAPI. AZ region is marked by white dotted lines. Images (**E**,**G**) are magnified regions of the central area of AZ containing vascular tissues from control and stressed plants, respectively. Abbreviations: AZ—abscission zone; D—distal region of AZ; P—proximal region of AZ; Ph—phloem vessels; VB—vascular bundle, X—xylem vessels. Bars = 40 µM.

Hydrolysis of the components of plant cell membranes, such as phospholipids, might be mediated by PLD and produce phosphatidic acid—a key molecule in the signaling pathways evoked by stress [52]. Furthermore, the activation of phospholipases (PLD or DAD1) leads to the formation of JA’s precursor—*α*-linolenic acid [53]. Thus, in the next step, we aimed to check the level and localization of PLD in flower AZ cells of drought-treated lupines (Figure 2). Western blot experiments showed the presence of two bands of ~90 and ~95 kDa, which were recognized by the anti-PLD antibody (Figure 2B). As densitometry analysis revealed, the first, smaller isoform was strongly accumulated in drought-treated AZ, while the second one was downregulated by stress and was abundant in the control variant (Figure 2A). We next used the same antibody to examine the cellular localization of PLD. We found that the response of these cells to stress was accompanied by the appearance of this enzyme in the whole AZ area, including the distal and proximal region (Figure 2F). Such labeling has not been observed in the control section (Figure 2D). When we looked at a higher magnification, strong, green fluorescence was emitted by vascular bundles located at the central area of drought-stressed AZ (Figure 2G). This region of the control section was characterized only by several luminous spots in the phloem and higher fluorescence emitted by the peripheral regions of xylem cells (Figure 2E).

### 2.3. Drought-Evoked Upregulation of Lipoxygenase (LOX)

**Figure 3:**
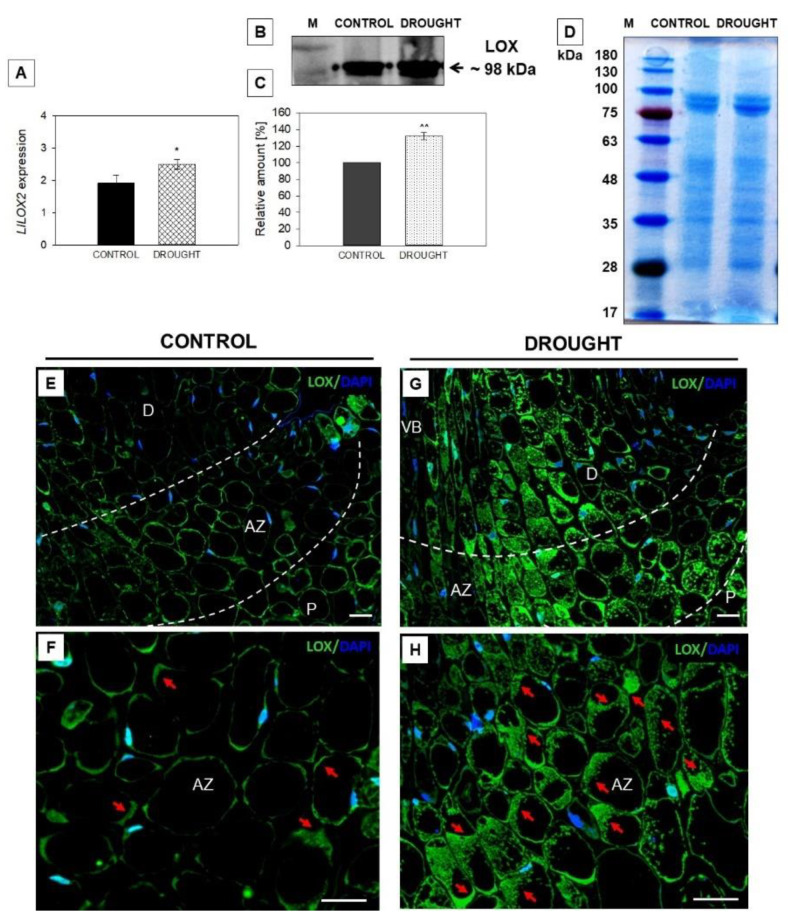
Lipoxygenase (LOX) is regulated by drought in the flower abscission zone (AZ) of *Lupinus luteus*. Analyses were carried out on flower AZs collected from 48 day-old lupines cultivated under drought (25% water holding capacity, WHC) or control (70% WHC) conditions. See Material and Methods for the details. Plant tissues were used for the expression analysis of *LlLOX2* (**A**). Asterix indicates a significant difference with * *p* < 0.05. The isoform of LOX of ~98 kDa was obtained on the nitrocellulose membrane in the Western blot reaction with an anti-LOX antibody (**B**). A band was scanned and quantitative densitometric analysis was made (**C**, 100% was set for the control). ^^^^ *p* < 0.01 indicates a significant difference. SDS-PAGE electrophoresis gel stained with Coomassie Brilliant Blue (**D**). Sizes of molecular mass marker (M) are shown in kDa. Immunodetection of LOX in the flower’ AZ of control (**E**,**F**) and drought-stressed (**G**,**H**) plants. Green fluorescence indicates LOX accumulation (marked by red arrows), while blue labeling corresponds to nuclei stained with DAPI. AZ area is marked by white dotted lines. Images F and H are magnified regions of AZ from control and stressed tissues. Abbreviations: AZ—abscission zone; D—distal region of AZ; P—proximal region of AZ; VB—vascular bundle. Bars = 40 µM.

Changes in membrane lipid metabolism could be related to the action of LOX, which catalyzes lipid peroxidation and affects lipid bilayer fluidity and permeability through the increasing lipid unsaturation [54]. So, we analyzed the influence of soil drought on the expression, protein level, and localization of LOX in lupine flowers’ AZ (Figure 3). The mRNA content of *LlLOX2*, which was identified recently by us and shown to be involved in AZ functioning [22], increased under water deficit conditions in flower AZ cells (Figure 3A). At the same time, we detected the isoform of LOX protein of ~98 kDa, accumulated strongly in the AZ under drought (Figure 3B,C). In the next step, the cellular localization of LOX protein was performed by using the same antibody as for the Western blotting. A green fluorescence labeling, indicating LOX presence, was detected in the whole region of AZ from flowers subjected to drought, in both proximal and distal areas (Figure 3G). At higher magnifications, strong fluorescence was observed in the cytoplasm region around the vacuoles of AZ cells (Figure 3H). The cells were almost filled with LOX protein. Such extensive staining was not seen in the control section (Figure 3E). Those AZ fragments were characterized by weak labeling, which presented in the peripheral areas of cells (Figure 3F).

### 2.4. The Level and Localization of Jasmonic Acid (JA), JASMONATE RESISTANT1 (JAR1), and CORONATINE INSENSITIVE 1 (COI1) in the Floral Abscission Zone (AZ) Are Affected under Drought Stress

**Figure 4:**
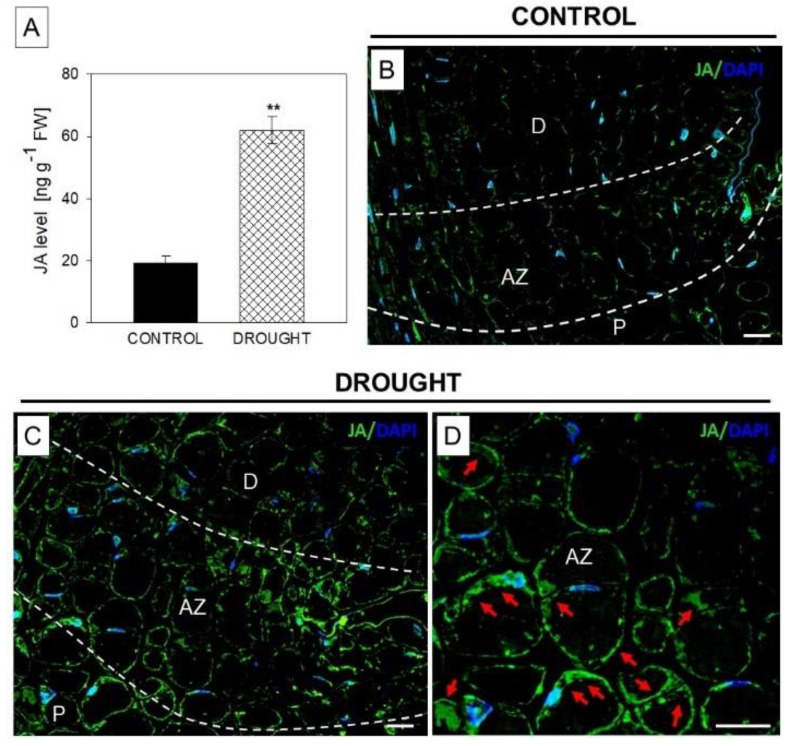
Drought upregulates the level of jasmonic acid (JA) in the abscission zone (AZ) of yellow lupine flowers. AZ fragments were collected for these analyses from 48 day-old plants subjected to drought (25% water holding capacity, WHC) or lupines cultivated in the soil of optimal moisture (70% WHC, control). Details in the Material and Methods section. The level of JA was examined using GC-MS (**A**). A significant difference is ** *p* < 0.01. Immunolocalization of JA in the AZ of control (**B**) or stressed (**C**,**D**) plants. Green fluorescence is related to JA localization (indicated by red arrows), whereas blue color corresponds to DAPI-stained nuclei. AZ area is marked by white dotted lines (**B**,**C**). (**D**) image is magnified AZ area presented on C. Abbreviations: AZ—abscission zone; D—distal region of AZ; P—proximal region of AZ. Bars = 40 µM.

In the subsequent step, we used chromatography and immunofluorescent methods to detect JA in flower AZ cells following drought (Figure 4). Our data revealed that this stress increased the level of JA almost three times when compared to the material collected from plants growing under optimal moisture (Figure 4A). After this, we used a specific antibody against JA to check the cellular distribution of this phytohormone. When lupine was subjected to drought, we observed green labeling, indicating JA’ presence in the proximal, distal, and whole AZ region (Figure 4C). Higher magnifications provided additional details about the subcellular localization of JA. It was located in the cytosolic regions of AZ cells, mainly around vacuoles and cellular membranes (Figure 4D). In the control section, the reaction did not show such strong labeling (Figure 4B).

**Figure 5:**
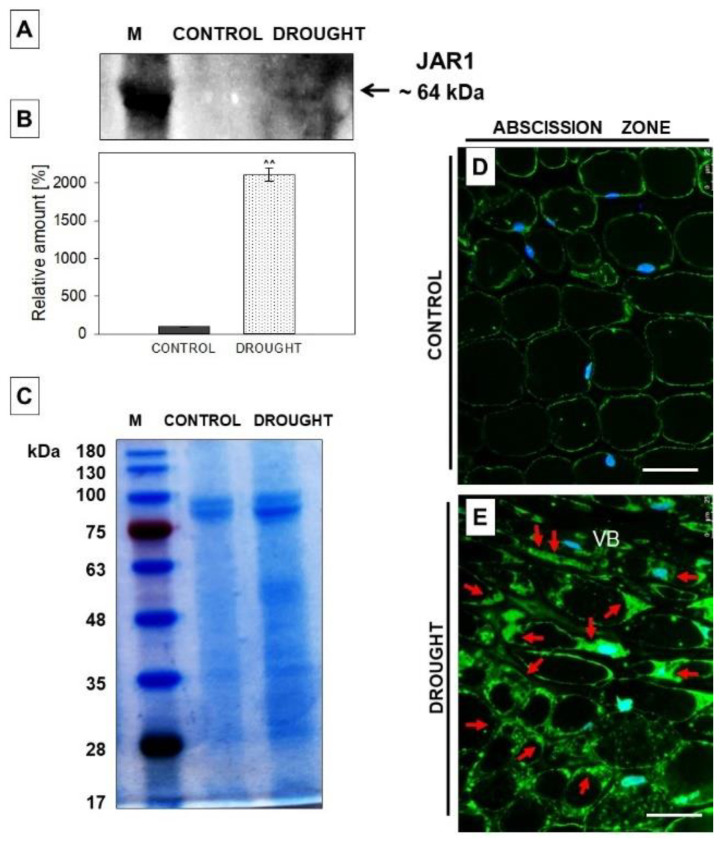
JASMONATE RESISTANT1 (JAR1) is induced in the abscission zone (AZ) cells of yellow lupine flowers in response to soil drought. AZ from flowers collected from 48 day-old plants growing under optimal soil moisture (70% water holding capacity, WHC) was a control, while the “drought” variant was AZ excised from 48 day-old plants subjected to drought (25% WHC), as written precisely in the Material and Methods section. Western blot analysis was performed with a JAR1 antibody and revealed the presence of a reactive band of ~64 kDa (**A**). The membrane was scanned, densitometry analysis was performed (100% was set for the control), and presented in (**B**). Protein samples separated by SDS-PAGE and stained with Coomassie Brilliant Blue (**C**). Protein marker (M) is displayed on the left and sizes in kDa are provided. Immunolocalization of JAR1 in flower AZ cells during soil water deficit (**E**) and under control conditions (**D**). Green fluorescence indicates JAR1 localization (marked by red arrows). Blue labeling corresponds to DAPI-stained nuclei. AZ region is restricted by white dotted lines. Abbreviation: VB—vascular bundle. Bars = 40 µM. A significant difference is ^^^^ *p* < 0.01.

To analyze the potential correlation of JAR (responsible for JA conversion into its various conjugates) and drought-evoked abscission in lupine, we used an anti-JAR1 antibody for Western blotting and immunofluorescent localization of this enzyme (Figure 5). One band of ~64 kDa reactive to the antibody was detected, and, importantly, it was accumulated under drought in flower AZ tissues (Figure 5A). The level of this isoform was approximately twenty times higher when compared to control variant (Figure 5B). We then used the microscopy technique to check the cellular distribution of JAR1 in AZ cells. The presence of this enzyme in the control section was restricted to the thin layer of cytoplasm (Figure 5D). In turn, observation of drought-treated plants revealed a high concentration of JAR1 in the flower AZ, including the vascular tissue, which is located in the central region of AZ (Figure 5E). The signal was diffused within the whole AZ cells; protein-free areas are possibly vacuolar regions.

**Figure 6:**
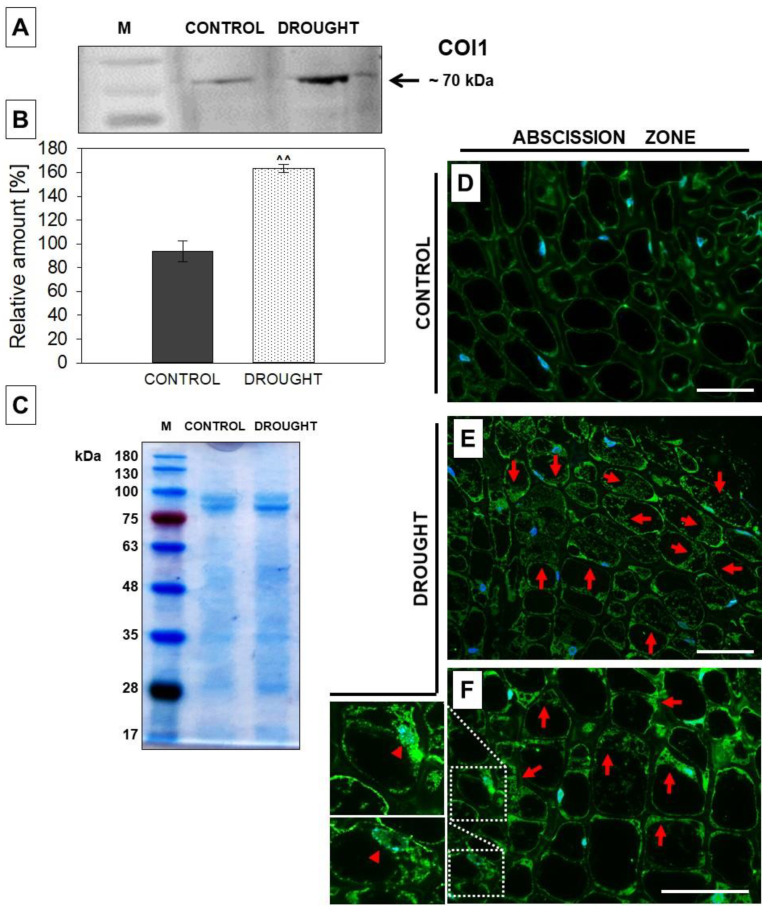
The content of coronatine insensitive 1 (COI1) in the flower abscission zone (AZ) of *Lupinus luteus* strongly increased under soil water deficit. Flower AZ was harvested from plants growing in the soil of optimal moisture (70% water holding capacity, WHC, control) or drought-stressed lupines (25% WHC). Detailed description in the Material and Methods section. Proteins were isolated and subjected to Western blot analysis with an anti-COI1 antibody, which revealed the presence of a band of ~70 kDa (**A**). Quantitative densitometry analysis was made and presented in (**B**) (100% was set for the control). ^^^^ *p* < 0.01 indicates a significant difference. Protein samples were separated by SDS-PAGE and stained with Coomassie Brilliant Blue (**C**). Mass marker (M) is provided on the left, sizes of proteins are displayed in kDa. COI1 antibody was used for immunolocalization analysis performed on control (**D**) and drought-stressed (**E**,**F**) tissues of AZ from flowers. A green fluorescence signal, indicated by red arrows, corresponds to the presence of COI1. Nuclei were detected by DAPI (blue color). Enlarged cells of stressed AZ are provided on the left side of the (**F**) image. These are magnified regions marked by white boxes and show colocalization of CO1 with nuclei (red arrowheads). Bars = 40 µM.

Jasmonate signaling can be mediated by the COI1 protein. We investigated the possibility of the contribution of this enzyme to drought-related events taking place in flower AZ. Firstly we applied an anti-COI1 antibody for the protein measurement using Western blot. One isoform of ~70 kDa appeared both in the control and drought-treated tissue (Figure 6A), however, the band showed higher intensity in the stressed AZ (Figure 6B). Immunofluorescent analysis revealed that COI1 was detected in almost every cell of the AZ region (Figure 6E). The fluorescent labeling was observed mainly in the nuclei (Figure 6F) and cytoplasm surrounding the central vacuole, as small fluorescent spots (Figure 6E). AZ from plants growing in optimal moisture was labeled as being much weaker; the signal was visible in the peripheral cellular regions (Figure 6D).

## 3. Discussion

The involvement of JAs in organ abscission has been already documented in the scientific literature, however, the changes mediated by these phytohormones directly in AZ have not been described so far. The first data regarding this issue in *L. luteus*, published recently, indicate that JAs are important components of the AZ cells’ functioning during the natural processes of flower abscission [22]. In turn, the presented paper is the first to investigate JAs-related events taking place in flower AZ under soil drought stress. The ROS formed during water deficit are signaling molecules that induce defense reactions in the plant [55]. However, the excess production of superoxide anion (O_2_^•−^) and hydrogen peroxide (H_2_O_2_) leads to disorders in the structure and function of tissues, including lipid peroxidation and electrolyte leakage, which is expressed by an increase in markers of oxidative stress, e.g., MDA [56]. The here-presented accumulation of MDA in flower AZ in *L. luteus* under water stress (Figure 1) indicates the lipid peroxidation state. These observations led us to suppose that drought in yellow lupine causes the destabilization of the lipid bilayer in flower AZ, which might involve the action of PLD and LOX. PLD is a key regulator of responses to abiotic and biotic stress, including drought [57]. Its activity is positively correlated with the sensitivity of plants to drought stress in cowpea, peanut and *Arabidopsis* [32,58,59]. The existence of various PLD isoforms, active in a different spatial and temporal manner during stress, reveals the complexity of regulation of various target proteins triggered in the PLD-dependent pathway and the production of lipid second messenger—phosphatidic acid (PA) [57]. The specific response of yellow lupine to soil drought stress involves the appearance in AZ cells of the PLD isoform of ~90 kDa (Figure 2B). Its level is much higher when compared to control AZ. In turn, the other PLD isoform of ~95 kDa presented in control variant was almost undetectable in drought stressed-AZ (Figure 2B). Such a specific relationship at the level of isoforms might suggest that one of them (~90 kDa) is required for normal cell functioning, while the second (~95 kDa), might play a role in the modification of the lipid bilayer under stressful conditions. Tissue-specific accumulation of PLD in response to drought was confirmed by immunocytochemical analyses (Figure 2F,G). This experiment has shown that the signal indicating PLD presence is low in control, however, the cell wall of the xylem emits green fluorescence, which corresponds to the unique localization of this enzyme (Figure 2), suggesting its potential role in extracellular signaling. A similar, xylem-specific presence of PLD was described in rice (OsPLDα1) [60]. On the other hand, PLD detected in the cytosol of drought-stressed AZ (Figure 2F) might mediate actin-dependent reorganization of the cytoskeleton, which has been previously pointed out in tomato [61], and *Arabidopsis* [62]. It is highly possible for this to happen in *L. luteus,* since we previously observed drought-evoked modification of the structure of AZ cells, e.g., cellular divisions, disruption of tissue integrity, and increased number of aggregates in the cytosol [21], which involves the modification of microfilaments in the cells. Furthermore, PLD participates in hormone signaling mediated by ABA [63], so its presence in the vascular bundles of lupine AZ under drought (Figure 2G) could be related to the accumulation of ABA, as we previously presented [21]. In line with these findings is the conception of Loveys [64], according to which ABA acts as a xylem-located signal formed under drought and transported to different plant’ parts.

Drought stimulates the expression and activity of enzymes responsible for the biosynthesis and signaling of JAs; among them, LOXs are sensitive to such conditions [55]. Here, we present that soil water deficit upregulates mRNA of *LlLOX2* and the level of the LOX isoform of ~98 kDa in flower AZ (Figure 3B). This is reflected by the tissue localization of LOX (Figure 3G,H). We recently observed a similar correlation following the natural activation of lupine AZ [22]. However, in this case, the accumulation of three isoforms (~98, ~100, and ~100 kDa) was noted. In combination with the findings from our previous work and this study, we can conclude that the isoform of ~98 kDa is highly sensitive to drought. This kind of stress upregulates *LOX1* in pepper [33] and *Agrostis stolonifera* [40], while upregulating *LOX2* in *Phaseolus vulgaris* [34]. Interestingly, a positive correlation between *LOX* expression and JA level was observed in *Triticum* [41] and *P. vulgaris* [34], indicating LOX as being crucial for maintaining the proper biosynthesis rate of these phytohormones. Furthermore, based on the data presented here, we supposed that soil drought causes destabilization of the lipid bilayer in flower AZ cells of yellow lupine, while changes in the occurrence and localization of enzymes related to this process—PLD and LOX (Figure 2 and Figure 3)—indicate that it may be related to JAs. Thus, we checked whether these modifications are accompanied by JA production. Cells of flower AZ from lupine accumulate high amounts of this hormone when the plant is subjected to drought (Figure 4A,C,D). So far, the positive impact of drought on JA production has been shown, however, to the best of our knowledge, the here-presented results comprise the first report regarding JA presence in organ AZ under stressful conditions. JA content is not stable under the influence of stress. For instance, water stress leads to the transient production of JA in soybean leaves within 2 h, and then its downregulation to control values by 4 h [65]. In turn, 5 days after drought in three *Arabidopsis* ecotypes (Ws, Ler, and Col-0), JA remains at basal levels, while a precursor’s (12-OPDA) content is induced [66]. Interestingly, drought-tolerant apricot transiently accumulates JA in leaves, which subsequently promotes their senescence to avoid further water loss by the plant [67]. Our current results could coincide with these reports. JA presented in the AZ cells of lupine flowers (Figure 4) might be responsible for the activation of abscission and flower separation, leading to increased water accessibility for other, developing organs. This hypothesis is highly possible given our recent findings suggesting that exogenous JAs applied directly to lupine flower AZ stimulates their abortion, and that the natural abscission of flowers is accompanied by a remarkable accumulation of JA in AZ cells [22].

Apart from JA, its derivatives are also characterized by physiological activity in response to stress. Application of MeJA induces drought-responsive genes, in turn, drought accelerates JAs biosynthesis including JA-Ile [68,69,70,71]. JA derivatives are formed in the reaction catalyzed by JAR. It has been proven that recombinant JAR1 can form JA conjugates with Ile, Leu, Val, Phe, and ACC (1-aminocyclopropane-1-carboxylic acid)—a precursor of ethylene [72,73]. *Arabidopsis* *jar1-11* displayed hypersensitivity to drought, while drought treatment of transgenic plants overexpressing *JAR1,* results in reduced wilting due to the saving of higher amounts of water [74]. Our methodology, Western blotting supported by a precise cellular localization, was used for the first time to visualize the occurrence of JAR1 directly in the AZ of the organ (Figure 5). The changing presence (Figure 5A) and localization of JAR in lupine AZ (Figure 5E) suggest that the response of these cells to drought is associated with JA conjugation.

The action of JAs as phytohormones and a proper physiological response of plants is determined by the tissue competence, related to the presence of the receptor. Currently, the only known receptor for JAs is COI1, which is a component of E3 ubiquitin ligase SCF^COI1^ and acts as a crucial player of the machinery involved in the perception of JAs [75]. The here-observed accumulation of COI1 in the lupine AZ of flowers (Figure 6A) might support the induction of the JA signaling pathway when the plant is subjected to drought stress. A consequence of these events is flower abscission, as we have recently shown [21]. The presence of COI1 in the cytosolic region (Figure 6E,F) could be an effect of the synthesis of this protein, while nuclear localization (Figure 6F) provides evidence for the action of COI1. It is known that once JA-Ile is accumulated in the cytosol, then it is transported to the nucleus via JASMONATE TRANSPORTER 1 (JAT1, an ABC transporter) [76] and binds to COI1, which triggers degradation of the repressors—JAZ [77]. Nevertheless, another scenario related to the distinct action of COI1 is possible, given the recent finding of [78]. According to the authors, COI1 can act independently of JA-Ile and modify the transcriptional activity of genes in an independent way, other than the JA signaling pathway. Thus, COI1 was suggested to be a candidate for a new class of “moonlighting protein” [78]. In the presented paper we provide results of JAR1 and COI1 levels and localization, which is a future perspective for further studies regarding the role of JAs’ derivatives under stress conditions.

## 4. Material and Methods

### 4.1. Plant Material and Growth Conditions

The plant material used was abscission zone (AZ) sections, dissected from the flower bases of yellow lupine (*Lupinus luteus* L.). Taper variety was sown as previously described [79]. Drought conditions were supplied similarly as in our recent papers [21,80]. In brief, plants were cultivated for 5 weeks, maintaining 70% water holding capacity (WHC). Then, part of the plants was watered to ensure 25% WHC for 2 weeks (drought variant), while the amount of water given for well-watered plants was unchanged (control variant). At the generative stage (48th day of development), flower AZ fragments were dissected using a razor 1 mm above and 1 mm below the AZ, those being the distal and proximal parts of the AZ, respectively (see Supplementary Material). For AZ excision and collection, we applied the procedure of [79]. The material was frozen in liquid nitrogen and stored at −80 °C for further RNA isolation, MDA analysis, protein isolation, and JA quantification. In turn, fresh sections were fixed immediately for microscopy assays. All experiments were performed in three replicates.

### 4.2. RNA Extraction and RT-qPCR

Frozen AZs samples (100 mg) were ground in liquid nitrogen using the ISOLATE II RNA Plant Kit (Bioline, UK) according to the manufacturer’s instructions. Isolated total RNA (1 µg) was used for cDNA synthesis with Transcriptor First Strand (ROCHE Diagnostics GmbH, Mannheim, Germany). The obtained cDNA was used for the analysis of *LlLOX2* (identified recently by Kućko et al. [22]) and *LlACT* (reference gene), performed with primers and hydrolysis probes (Supplementary Table S1). Reactions were carried out in a LightCycler 2.0 Carousel-Based System (ROCHE Diagnostics GmbH, Mannheim, Germany), using a LightCycler TaqMan Master Kit (ROCHE Diagnostics GmbH, Mannheim, Germany) following the previous procedures optimized for the lupine flower AZ [21,81,82].

### 4.3. Material Fixation and Immunocytochemical Assay

For microscopy analysis, fresh AZ sections were fixed in the solution of 4% paraformaldehyde mixed with 0.25% glutaraldehyde in 1 × PBS buffer pH 7.2, supplemented with 1% N-(3-Dimethylaminopropyl)-N′-ethylcarbodiimide hydrochloride (Sigma-Aldrich, St. Louis, MO, USA) overnight at 4 °C. Further steps of dehydration, resin embedding, and polymerization have been applied, following our standard procedure for lupine tissues described by Wilmowicz et al. [81]. Necessary chemicals were provided by Sigma-Aldrich, St. Louis, MO, USA. Tissues were cut to the sections (1.5 μm) using an ultramicrotome (UTC Ultracut Microtome, Leica, Wetzlar, Germany) and put onto the glass slides. Detection reactions were carried out according to Wilmowicz et al. [81] by incubating sections with primary antibodies provided by Agrisera (Vännäs, Sweden) against JA (AS11 1799), LOX (AS06 128), PLD (AS09 556), JAR1 (AS16 3688), and COI1 (AS12 2637), diluted 1:50 in 1% bovine serum albumin (BSA) in PBS, pH 7.2. After overnight incubation (4 °C), sections were washed with 1 × PBS buffer pH 7.2 and secondary antibodies (anti-rabbit DyLight IgG Alexa Fluor 488-conjugated IgG, AS09 633, Agrisera, Vännäs, Sweden), diluted in 1 × PBS buffer at pH 7.2, were applied for 2 h (37 °C). The results were documented using a fluorescent microscope (DM6000B, Leica, Wetzlar, Germany).

### 4.4. Jasmonic Acid Detection with GC-MS

For JA analysis, the methodology of Gundlach et al. [83] and Fan et al. [84], with some modifications [22], was applied. All chemicals were provided by Sigma-Aldrich, St. Louis, MO, USA. AZ fragments (~0.5 g) were homogenized in liquid nitrogen. JA was extracted twice in 40 mL 90% (*v*/*v*) MeOH and, subsequently, internal standard (250 ng d_2_-MeJA was added). The extract was reduced to the water phase, acidified to pH 2.0 with 12 M HCl, and centrifuged (12,000× *g*, 30 min). The obtained supernatant was supplemented twice with 20 mL of chloroform and mixed for 10 min. Collected organic phases were dried, while the obtained pellet was dissolved in 3 mL of n-hexane and extracted with solid-phase extraction SPE method (Backer-bound SPE silica gel, 500 mg, 3 mL; J.T. Backer, Philipsburg, NJ, USA). The column was firstly washed with 5 mL n-hexane and then eluted with 5 mL of n-hexane:diethyl ether, 1:2 (*v*/*v*) with 0.5% (*v*/*v*) acetic acid. After that, the sample was evaporated, dissolved in 50 µL of MeOH, and samples were analyzed by GC-MS-SIM (Auto-System XL coupled to a Turbo Mass, Perkin Elmer, Waltham, MA, USA) using a DB-5 column (30 m × 0.25 mm, 0.5 μm phase thickness). The following temperature program was applied: 80 °C for 1 min, 80–160 °C at 10 °C/min, 160–230 °C (5 °C/min), a flow rate of 1 mL/min, and an injection port temperature of 250 °C. GC/MS-selected ion monitoring was performed by monitoring 193, 195, 198, 224, 226, and 229 m/z. After that, the samples were methylated using diazomethane, evaporated, dissolved in 50 µL of MeOH, and analyzed by GC-MS-SIM one more time. The level of JA was analyzed based on the difference of the area of the ions obtained from the GC-MS analysis after methylation and for the same ones before methylation.

### 4.5. MDA Determination

For MDA analysis we followed Hodges et al. [85] methodology by using thiobarbituric acid (TBA). Minor modifications described in our previous paper [22] were applied.

### 4.6. Western Blotting

Frozen AZs sections (~0.4 g) were powdered in liquid nitrogen and mixed with 1 mL extraction buffer (50 mM Tris-HCl, pH 8.0, 300 mM NaCl, 10% *v/v* glycerol, 1 mM EDTA). Samples were centrifuged (4 °C, 10 min). All necessary chemicals were provided by Sigma-Aldrich, St. Louis, MO, USA. The protein-enriched supernatant was stored at −20 °C and used for further analyses. The total pool of proteins was estimated with the Bradford method [86]. An amount of 25 µg of denatured proteins and molecular mass marker (BlueStar Prestained Protein Marker, Nippon Genetics) were separated on two 4–12% (*w*/*v*) polyacrylamide gels (SDS-PAGE) using a Criterion^TM^ Cell apparatus (Bio-Rad, Hercules, CA, USA). One gel was stained with Coomassie Brilliant Blue, while the second was subjected to electroblotting onto the nitrocellulose membrane. This membrane was blocked firstly with 1% BSA solution in TBS buffer at pH 7.5 for 1 h, at room temperature. After that, the membrane was incubated at 4 °C overnight with an anti-PLD antibody (AS09 556, Agrisera, Vännäs, Sweden) diluted at 1:1000 in 0.5% BSA solution in TBS buffer, pH 7.5. Then, the membrane was washed 3 times with TBS buffer pH 7.5 and probed with DyLight 488-conjugated anti-rabbit IgG (AS09 633, Agrisera, Vännäs, Sweden) diluted 1:10,000 for 2 h at room temperature. Antigen was detected by using ECL SuperBright (AS16 ECL-SN, Agrisera, Vännäs, Sweden) and the signal was visualized using a ChemiDoc™ Touch Imaging System. The same procedure was applied for Western blotting with anti-LOX antibody (AS06 128, Agrisera, Vännäs, Sweden), anti-JAR1 antibody (AS16 3688), and anti-COI1 antibody (AS12 2637). For all bands on the membranes, image processing software (ImageJ) was used for densitometry analyses. Average densitometry values were presented on charts.

### 4.7. Statistical Analysis

The obtained data are results of the analysis performed in three biological replicates, each biological sample was examined two times (*n* = 3). The data represent the mean ± standard error (SE). Statistical analyses and charts were made using Microsoft Excel and SigmaPlot 2001 v.7.0. (Chicago, IL, USA).

## 5. Conclusions

To summarize, oxidative stress generated by drought causes the peroxidation of lipid compounds of membranes, which are then used for reactions catalyzed by PLD and LOX, leading to the formation of fatty acid-derived plant hormone—JAs—in flower AZ cells. As a consequence of JA-mediated AZ activation, a flower is then cut off. We have provided drought-specific molecules toward a better understanding of the physiological process underlying the response of flower AZ to water deficits in soil.

## Supplementary Materials

The following supporting information can be downloaded at: https://www.mdpi.com/article/10.3390/plants11040527/s1. Figure S1: Histological analyses of abscission zone (AZ) from flowers of *Lupinus luteus*. Figure S2. The results of control immunofluorescent reactions performed with omitting primary antibodies. Table S1. Specific primers and probes used in reactions.

## Abbreviations

AZ—abscission zone, COI1—CORONATINE INSENSITIVE1, GC-MS—gas chromatography mass spectrometry, JA—jasmonic acid, JAR1—JASMONATE RESISTANT1, JA-Ile—jasmonoyl-L-isoleucine, LOX—lipoxygenase, MDA—malondialdehyde, MeJA—methyl jasmonate, PLD—phospholipase D, PUFA—polyunsaturated fatty acids, ROS—reactive oxygen species.
