# Supplementary material for: Jasmonate-Dependent Response of the Flower Abscission Zone Cells to Drought in Yellow Lupine
Source: Plants (Basel). 2022 Feb 15;11(4):527. doi: 10.3390/plants11040527 (PMC8877524; doi:10.3390/plants11040527)
Supplement: Supplementary file 1 [file plants-11-00527-s001.zip › plants-1594362-supplementary.pdf]

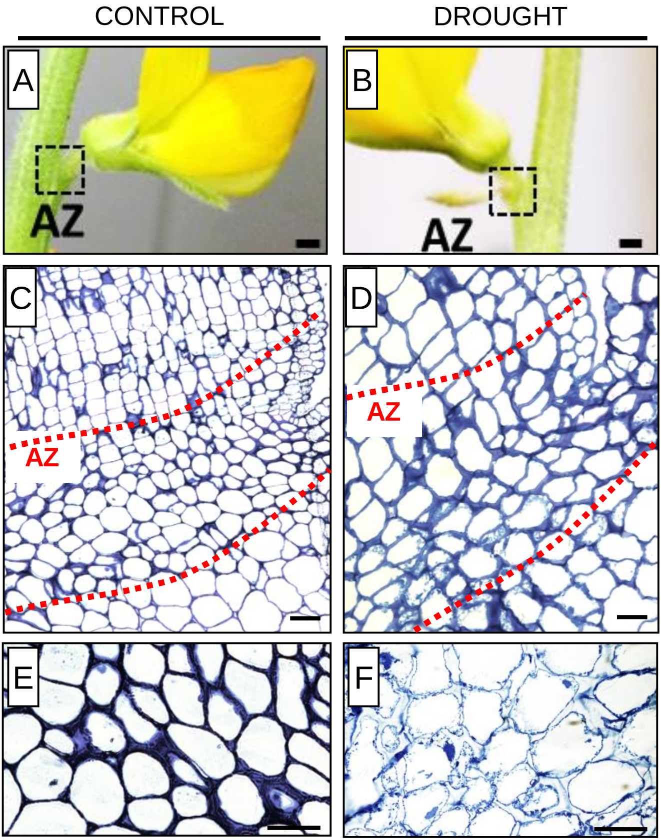

Supplementary Figure S1. Histological analyses of abscission zone (AZ) from flowers of *Lupinus luteus.* AZ fragments were excised from plants cultivated for 2 weeks in drought (25% water holding capacity, WHC) (B) or under optimal moisture conditions (70% WHC) (A). Sections of control (C) and stressed (D) AZ stained with toluidine blue. Magnification of AZ cells from control (E) and stressed (F) plants. Abbreviations: AZ – abscission zone. Bar - 40 µm.

Supplementary Table S1. Specific primers and probes used in reactions.
F, forward primer; R, reverse primer

Gene	Primer sequence 5'-3'	UPL probe no.	Product size (bp)
LlLOX2	F: TTTTCAACACTGGGCTTGACT<br>R: CTCGGCATGATGGCTTCTA	133	70
LlACT	F: TAATGGTTGGGATGGGTCAG<br>R: CAAGGTGAGAATACCCCTCT	165	74

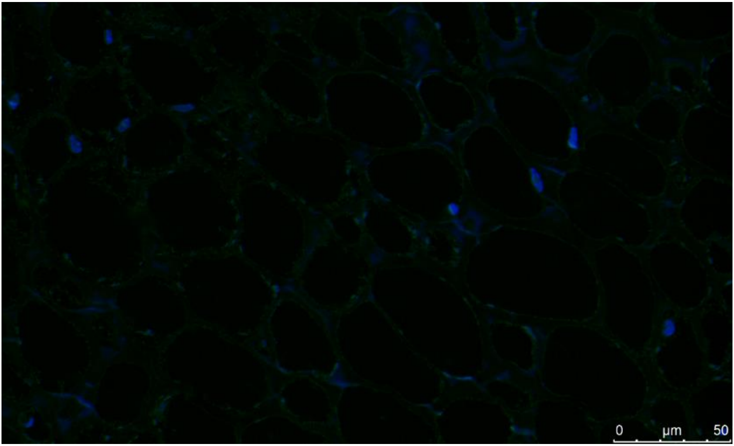

Supplementary Figure S2. The results of control immunofluorescent reactions performed with omitting primary antibodies. Controls were performed using secondary antibodies only. Rabbit IgG DyLight 488 conjugated (Agrisera, Sweden).
